# Changing Polygenic Penetrance on Phenotypes in the 20^th^ Century Among Adults in the US Population

**DOI:** 10.1038/srep30348

**Published:** 2016-07-26

**Authors:** Dalton Conley, Thomas M. Laidley, Jason D. Boardman, Benjamin W. Domingue

**Affiliations:** 1 Department of Sociology, Princeton University, Princeton, NJ 08644, USA; 2 Department of Sociology, New York University, New York, NY 10012, USA; 3 Institute of Behavioral Science and Department of Sociology, University of Colorado, Boulder, CO 80309, USA; 4 Graduate School of Education, Stanford University, Stanford CA94305, USA.

## Abstract

This study evaluates changes in genetic penetrance—defined as the association between an additive polygenic score and its associated phenotype—across birth cohorts. Situating our analysis within recent historical trends in the U.S., we show that, while height and BMI show increasing genotypic penetrance over the course of 20^th^ Century, education and heart disease show declining genotypic effects. Meanwhile, we find genotypic penetrance to be historically stable with respect to depression. Our findings help inform our understanding of how the genetic and environmental landscape of American society has changed over the past century, and have implications for research which models gene-environment (GxE) interactions, as well as polygenic score calculations in consortia studies that include multiple birth cohorts.

This study evaluates changes in polygenic penetrance—defined as the association between a polygenic score (PGS) and its associated phenotype—across recent birth cohorts in the United States. The answer to this question informs our understanding of how the genetic and environmental landscape of American society has changed over the past century, and offers suggestive evidence for the selective influence of environment on genetic expression. Our findings also have important implications for PGS calculations in consortia studies that include multiple birth cohorts. This inquiry would not have been possible even a decade ago, before the development of PGS techniques to predict complex phenotypes^1^. The approach is not without its limitations; however, the scalar variables provided by PGS construction are unique in that they allow researchers to ask a number of questions that were not possible with latent heritability models. This allows for fresh opportunities to explore a range of issues, from polygenicity of traits to gene-environment (GxE) interactions.

In the present paper, we exploit this opportunity by asking whether the associations between PGS and several phenotypes have changed over the course of the 20^th^ century in the U.S. Because the economic, social, and physical environments underwent dramatic changes during this period, it is likely that the association between a PGS and its related phenotype has also evolved as a consequence^2^. We examine five important phenotypes—height, body mass index (BMI), education, depression, and heart disease—chosen due to their key associations with health and mortality, the different age ranges at which they are salient^3^,^4^,^5^, and the fact that GWAS results (for all SNPs and not just top hits) are available for all five^6^,^7^,^8^,^9^,^10^. We find that while height and BMI show increasing PGS penetrance over the course of the 20^th^ century birth cohorts, education and heart disease exhibit the opposite trend. In contrast, the association between depression and its underlying genetic architecture remained stable over the same period.

Additive heritability (for which PGS penetrance is a proxy), independently of how it is measured, is contingent on the social structure. Indeed, heritability is not a fixed parameter across time and place but is always a ‘local perturbation analysis'^11^. Supposing a phenotype to be the product of a complex process involving both genetics, environment, and perhaps their interactions (that is, *y*_*i*_ = *f*(*G*_*i*_, *E*_*i*_) + *ε*_*i*_), a complete analysis would require that we first know the partial derivatives of the unknown function *f*(G, E). Absent a specified model of *f*(G, E), many scholars, particularly in the social sciences, have attempted to inductively model gene-environment correlations (rGE) and interactions (GxE). Starting with the seminal paper in this area of scholarship^12^, most of these studies rely on endogenous measures of environment and/or fail to adequately control for population structure, thereby producing under-identified results that may reflect rGE, GxE, ExE or GxG^13^.

A few exceptions to this trend include studies that deploy nationally-representative, genome-wide data with controls for principle components in order to address population stratification on the genetic side while econometrically exploiting natural experiments on the environmental side to assure exogeneity of environment^14^. A promising avenue in this regard has been scholarship that takes advantage of data spanning a wide range of birth cohorts to assess how heritability may be changing over the shifting (if unmeasured) environment across decades. For instance, recent research has shown that a PGS for physiological predisposition to tobacco use has exhibited more robust correlations over time with phenotypic measures of smoking in the U.S. population^15^. Studies which employ sibling and twin comparisons and candidate gene studies show the same pattern of increasing genetic penetrance with respect to tobacco use among recent cohorts^16^,^17^. These results suggest that as the dangers of tobacco use were publicized in the latter half of the 20^th^ century, the underlying genotype signifying a greater propensity to smoke exerted a more pronounced influence on behavior.

Other research shows a similar historical shift in genomic influence on physical characteristics, with increasing associations between genetic architecture and BMI in recent decades for US adults^18^,^19^. Likewise, twin-based models of the heritability of education appear to show an increasing effect of genotype over a similar time period^20^. We expand on this literature by focusing on a wider breadth of phenotypes and employ polygenic scores based on millions of SNPs rather than individual markers in identifying historical shifts in genetic expression.

Some have argued that these changes reflect the relative increase of genetic over social factors as determinants of complex behavioral traits like smoking, rather than a true increase in the causal association between genetic polymorphisms and phenotypes. This distinction is important because it emphasizes genetic penetrance rather than expression, per se. That is, the social and historical context can, at times, mask small genetic associations because the environment may be ‘pushing’ the phenotype, which limits our ability to observe penetrance^16^. The social environment can also serve as a trigger (or, alternatively, as a controlling influence) in which differential rates of expression (or methylation) in response to specific environmental signals denotes a biological mechanism, through which the environment causes genes to function in a particular manner^21^.

## Results

We used data from the Health and Retirement Study (HRS). Details about inclusion in the sample and selective attrition can be found in the Supplementary Information notes. Our data are from the 2012 wave of the HRS, and allowed us to observe the consistency of PGS-phenotype correlations across birth cohorts in the mid-20^th^ century among U.S. adults. Respondents were born between 1919 and 1955 and, on average, went on to complete over 13 years of education. Nearly 40% of the respondents self-reported heart disease. Baseline associations between the five traits and their respective polygenic scores (Supplemental Table S2) are significant at conventional alpha levels. The polygenic score for BMI is the best predictor of its associated outcome, followed by education and height.

**Figure 1:**
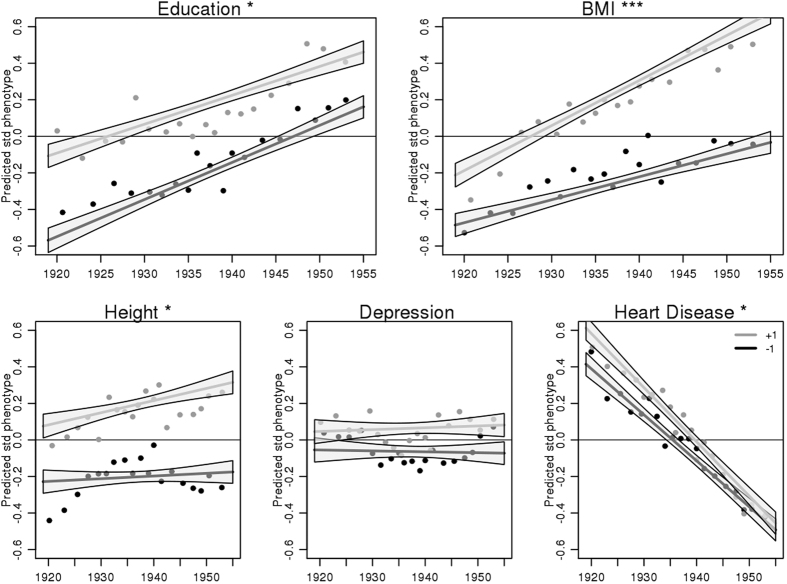
Predicted standardized values of selected phenotypes by polygenic score (+1 or −1 standard deviations), across birth cohorts among genotyped respondents in the Health and Retirement Study (N = 8,865). Height (p < 0.05) and BMI (p < 0.001) polygenic scores become more predictive in later birth cohorts while education (p < 0.05) and heart disease (p < 0.05) PGSs become less predictive. Depression does not show a significant trend. The lines show fitted values for those at 1 SD above (gray) and below (black) the mean. Points are based on binned means for two groups of respondents (standardized value below 0, black; standardized value above 0, dark gray). For each group, the distribution of birth years is divided into 20 subgroups with approximately equal numbers. Plotted points are the mean birth year and response for these subgroups.

We interacted the PGS for each trait with birth year to predict the corresponding phenotypes in Fig. 1 (model also included main effects for both birth year and phenotype; see Equation 2 in Methods). We find that, while there is tendency for those in later birth cohorts to accrue more education, the predictive power of genotype for education is declining over time. This finding is contrary to some twin-based evidence that the genetic penetrance for education has risen^20^; this could be due to a number of dynamics including the inherent differences between twin methods and the PGS approach, differences in the birth cohorts studied or changing gender dynamics. (We discuss potential difference sand explanations in depth in the SI on pages 10–11). Similarly, declines in heart disease are matched by declines in the predictive natures of the heart disease PGS. Meanwhile, the predictive power of height and BMI polygenic scores have increased significantly, while depression appears flat. Our results showing an increased PGS penetrance of BMI in particular among more recent cohorts of Americans are broadly consistent with recent researchbased on a more limited polygenic score and other forms of genetic analysis^18^,^19^.

One potential explanation for these trends in PGS penetrance could be due to changes in the genetic variation in the population that could result from differential fertility and/or genetic assortative mating^22^,^23^,^24^. To assess this latter possibility, we calculated the variance for each of the five PGSs across birth cohorts. These are reported in the Supplemental Information Fig. S5, Panel B. For all the scores, variances are unchanged across birth cohorts, supporting the understanding that changes in PGS predictive power reflect GxE effects that result from a shifting environmental landscape. Namely, if the variance component for G is unchanged, any change in additive heritability or SNP-based PGS prediction is likely due to a shift in the variance component for the environmental portion. We also perform other sensitivity checks related to mortality and sample ascertainment (presented in detail in the Supplementary Information), and find that our results broadly reflect a changing influence in environmental conditions, and do not appear to be driven by biases introduced by the data (see SI, Page 5–10). Likewise, our results become stronger when measurement error for each PGS is taken into consideration through SIMEX analysis (Table S3) and are robust to Huber-White adjustments for clustering by household (Table S4). That said, our power to detect the interaction term is limited for some phenotypes (particularly depression—see Table S2 and SI notes for discussion), so replication of our results will be important.

## Discussion

The twentieth century witnessed massive shifts in the social and nutritional environment of the United States. The change from an agrarian society to an industrial and post-industrial one has well documented effects on population health^25^ and is also associated with the expansion of schooling^26^, medical improvements^27^, increased longevity^28^, and caloric abundance^29^. Any or all of these changes may influence not only relationships between important phenotypes but between those phenotypes and their underlying genotypes as well. Under this multi-dimensionally shifting environmental regime, the genotypic effects of height and BMI PGSs evince trends of increasing predictive power, while education PGS shows a declining association with years of schooling, perhaps due to policy and structural changes in society that has reduced variation in the phenotype (see Panel B of SI Fig. S4).

As nutritional deprivation receded as a restraining force on genetic expression, height and weight could more “accurately” reflect underlying genetic potential as measured by common SNPs. Meanwhile, educational “abundance” had the opposite effect: with the steady expansion of schooling we find that rather than constraints on the full extent of ability being lifted to reveal increasing genetic penetrance, we observed declining genetic prediction among more recent cohorts. During this time, secondary schooling became nearly universal and post-secondary education more common, yet the genetic signal was weakened. Thus, in some cases—like height and BMI— environmental barriers can act to suppress genetic effects, while in others (such as education) such obstacles can act to accentuate genetic associations. This may be a useful dichotomous classification scheme to apply to cohort analysis of genetic influence on other phenotypes going forward.

## Materials and Methods

**Phenotypes** were computed based on RAND Fat Files, version N (which covers data collection up until 2012). We examined:

- Education: Total years of educational attainment.
- BMI: Mean BMI over all available waves.
- Height: Max height over all available waves.
- Heart Disease: Whether a respondent ever reports heart problems (rXheart).
- Depression: Mean CESD score over all available waves. This variable had a skewed distribution, so it was transformed via the logarithm (after adding one to everyone’s mean).

Sample descriptives are shown in Table S1.

## Methods

Polygenic Scores (PGSs) were first suggested in 2007 as flexible tools for quantifying the genetic contribution to a phenotype^30^. Polygenic scores have several attractive features. First, unlike candidate genes, they are “hypothesis-free” measures—i.e. ex ante knowledge about the biological processes involved is not needed to estimate a score for a particular phenotype. Rather, a polygenic scores casts a wide net across an individual’s entire genome to yield a single quantitative measure of genetic risk, or genetic risk score (GRS)^31^,^32^,^33^,^34^, allowing researchers to explore how genes operate within environments where the biological mechanisms are not yet fully understood^35^.

PGSs were constructed based on publicly available data from recent GWAS (additional details on the genetic data and the construction of polygenic scores are available in the SI)^6^,^7^,^8^,^9^,^10^. The same approach was conducted with each set of GWAS results. Briefly, SNPs in the HRS genetic database were matched to SNPs with reported results in a GWAS. Since the risk allele is not always readily identifiable, we removed all ambiguous SNPs. For each of these SNPs, a loading was calculated as the number of phenotypically associated reference alleles multiplied by the effect-size estimated in the original GWAS as shown in Equation 1, below. Thus, a polygenic score (PS) for individual 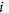
is a weighted average across the number of SNPs (*n*) of the number of reference alleles *x* (0, 1 or 2) at that SNP multiplied by the score for that SNP (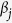):


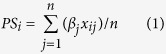


where SNPs with relatively large p-values will have small effects (and thus be down weighted in creating the composite), so we do not impose a p-value threshold. Loadings were summed across the SNP set to calculate the polygenic score. The score was then standardized to have a mean of 0 and SD of 1 for ease of interpretation (though analysis of raw scores does not change results). Genetic analyses were done using the second-generation PLINK software^36^. Finally, scores were residualized on the top 10 principal components computed from the non-Hispanic whites in HRS to ensure that none of the reported results are due to changes in population stratification (though results without residualization on PCs do not change, see Fig. S3 of SI). To examine changes in PGS penetrance, we estimated Equation 2:


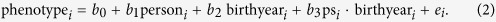


Huber-White correction for the non-independence of spousal pairs does not change results (see Supplementary Information Table S4).

## Additional Information

**How to cite this article**: Conley, D. *et al.* Changing Polygenic Penetrance on Phenotypes in the 20^th^ Century Among Adults in the US Population. *Sci. Rep.* **6**, 30348; doi: 10.1038/srep30348 (2016).

## Supplementary Material

Supplementary Information
