# Supplementary material for: Changing Polygenic Penetrance on Phenotypes in the 20th Century Among Adults in the US Population
Source: Sci Rep. 2016 Jul 26;6:30348. doi: 10.1038/srep30348 (PMC4960614; doi:10.1038/srep30348)
Supplement: Supplementary Information [file srep30348-s1.pdf]

Changing Polygenic Penetrance on Phenotypes in the 20th Century Among Adults in the US Population

Supplemental Information

Dalton Conley[1], Thomas Laidley[2], Jason D. Boardman[3], Benjamin W. Domingue[4]

Contents:

Supplementary Notes | 1

Supplementary Figures | 11

Supplementary Tables | 19

Supplementary References | 47

[1] Department of Sociology, Princeton University, Princeton, NJ 08644
[2] Department of Sociology, New York University, New York, NY 10012
[3] Department of Sociology, University of Colorado, Boulder, CO 80309
[4] Graduate School of Education, Stanford University, Stanford CA94305

Supplementary Information

Addition information on the analytic and genetic samples

Table S1 shows descriptive statistics for the entire sample of non-Hispanic white HRS respondents born between 1919 and 1955. Figure S1 shows the raw and residualized polygenic scores. In general, the polygenic scores have approximately normal distributions. Note that the density for height's polygenic score shows a local maxima around -3. Table S2 and Figure S2 show associations between polygenic scores and their associated phenotypes after residualizing on the top 10 PCs.

Information on Polygenic Score Construction

Genetic data used here for the HRS is based on DNA samples focus on single nucleotide polymorphisms (SNPs) collected in two phases. The first phase was collected via buccal swabs in 2006 using the QuiagenAutopure method. The second phase used saliva samples collected in 2008 and extracted with Oragene. Genotype calls were then made based on a clustering of both data sets using the Illumina HumanOmni2.5-4v1 array. SNPs are removed if they are missing in more than 5% of cases, have low MAF (0.01), and are not in HWE ($p<0.001$). We retained 1,741,345 SNPs after removing those which did not pass the QC filters.

The scores were constructed via a pipeline that we have previously used elsewhere[15,34]. (Note that the software used to construct the polygenic scores is available at: https://github.com/ben-domingue/HeritHelper.) We conducted the same basic process for each set of GWAS results[6-10]. First, SNPs in the HRS genetic database were matched to SNPs with reported results in the GWAS (note that we did not use imputed genotypes). Ambiguous SNPs, where the risk allele could not be readily identified, were dropped.The scores used here utilized substantial numbers of SNPs: BMI 838,490; height 837,087; depression 622,170; heart disease 807,011; education 771,589. For each of the remaining SNPs, a loading was calculated as the number of smoking associated alleles multiplied by the effect-size estimated in the original GWAS. SNPs with relatively large p-values will have small effects (and thus be down weighted in creating the composite), so we do not impose a p-value threshold. Loadings were summed across the SNP set to calculate the polygenic score (using the plink defaults for handling missing genotypes). The score was then standardized to have a mean of 0 and SD of 1. We

then residualized each score on the top 10 PCs (computed amongst the non-Hispanic whites in the HRS genetic sample).

Sensitivity Analyses

The left-hand side of Table S3 contains coefficient estimates from the main model presented in the paper (these are the basis for Figure 1 from main text). Here we consider sensitivity analyses related to three issues: measurement error in the polygenic score as a predictor of a phenotype, population stratification, and the fact that the analyses are based on spousal pairs in many cases. We find our results to be robust to each of these issues. We also consider our power for recovering time-varying effects.

Adjustment for measurement error

We used SIMEX to adjust for the fact that the polygenic score for each respondent is a noisy indicator of their true genetic risk[37]. This worked as follows:

1. We first estimate heritabilities via GCTA[38] for the four outcomes (4). Denote these h^2_{GCTA}.

2. We then assume that, for each outcome

$$y = g_t + e_1$$

whereg_t is the unobserved true polygenic score and is related to the heritability via the following expression:

$$h^2_{GCTA} = \frac{\mathrm{Var}(g_t)}{\mathrm{Var}(y)}.$$

We then assume that

$$g_o = g_t + e_2$$

Where g_o is the observed polygenic score. The error terms ($e_1$and e_2) are both assumed to be white-noise random errors (i.e. independent of g_t)

3. Usage of SIMEX requires an estimate for Var(e_2). We obtain that by first noting that

$$\mathrm{Var}(g_t) = h^2_{GCTA}\mathrm{Var}(y)$$

And then using

$$\mathrm{Var}(e_2) = \mathrm{Var}(g_o) - h^2_{GCTA}\mathrm{Var}(y)$$

by the assumption of independence with respect to e_2.

4. This estimate for $\mathrm{Var}(e_2)$ is then used in SIMEX to simulate predictors

$$g_S \sim \mathrm{Normal}[g_o, (1+\lambda)\mathrm{Var}(e_2)].$$

For different values of λ (typically over a grid between 0 and 2), a trend in the estimates of the relevant covariates is established which is then extrapolated back to the case where $\lambda = -1$. Under certain assumptions (e.g. additive measurement error) that are reasonable in the context of polygenic scores, this is the unbiased estimator.

Results are shown in the right-hand side of Table S3. The SIMEX-adjusted coefficients are uniformly larger in magnitude. The interaction coefficients are much stronger, at least 50% larger than their unadjusted baseline. As is to be expected, there is also an increase in the standard error associated with the parameter estimates after SIMEX.

Implications of Population stratification

Our approach in the main text includes a stringent correction for population stratification (i.e. residualizing on the PCs). This might be an overcorrection. Figure S3 is an updated version of Figure 1 from the main text based on polygenic scores that are not residualized for population stratification. A comparison of these figures suggests that none of the substantive findings change. For example, the coefficient for the education polygenic score interaction with birth cohort from Equation 2 in Figure 1 of main text is -0.00225 (SE=0.00113) while in Figure S3 it is -0.00213 (SE=0.00113).

Corrections for dependence

Table S4 compares regression estimates from Equation 2 before and after Huber-White corrections for the fact that spouses are nested within household in Equation 2. Key findings are robust to this correction.

Power Analysis

Table S2 displays the results of a post-hoc power analysis for our sample. In one column, we show the R^2 for the full GxE model (PGS, Cohort and PGS x Cohort) with the associated power to detect this effect size for the hypothesized model in the column to its right. All models have ~100 percent power to detect the R^2 of the size we are finding. To the right of this analysis are columns that show the partial R^2 for the transition from a main effects model (PGS, Cohort) to a full, GxE model as described above. Here we obtain a range of powers, with a low of 48% to a high of ~100%. Though 48% is obviously below the ideal of 80% power or even the 60% threshold, given the overall power of the hypothesized model (i.e. K=3), we feel that we have an adequate sample size to detect the statistically significant effects we report. Of course, the ultimate test is replication in another dataset.

Figure S4 graphically depicts results from a power analysis based on the main effects, sample size, and residual error variance of the main analysis and hypothesized interaction effects of -0.01 to 0.01 in increments of 0.005 (we conducted 1000 simulations for each increment). The black and red vertical lines represent observed estimate and SIMEX estimates (i.e. left and right sides of Table S3) respectively. The true interaction is, of course, unknown, but the evidence suggests that the observed interaction (black line) is too low due to attenuation associated with the measurement error of the polygenic score. Thus, we consider the SIMEX estimate as well and use these two points to suggest a range of reasonable hypothetical effects.

We are clearly well powered to detect BMI effects in the range of hypothesized effects. For other phenotypes, our power varies. For education, power varies from ~0.5 to 0.9 over the range of hypothetical effect estimates. Fairly comparable results hold for height and heart disease. We are very poorly powered to detect interactions for temporal changes in the genetic influence on depression.

A discussion of higher order concerns

In the following sections, we consider several potential higher-order confounders to the analyses discussed here. These concerns are not issues that can be addressed via straightforward sensitivity analyses, so we focus on simulations and relevant empirical evidence to suggest the degree to which they are potential confounders.

Mortality Bias and Changing Genotypic Penetrance

One potential concern with this exercise is mortality bias. Since HRS respondents had to survive until the 2000s in order to be included in the sample with genotype and phenotype information, the earlier cohorts in our analysis include individuals who lived longer on average.   Distinguishing sample selection from age and cohort effects is not an easy task, however. For example, in Figure S5 (Panel A), below, we see that there is a steep increase in the mean education by body mass index and birth cohort, with the later-born groups having much higher average years of schooling and BMI than the earlier-borngroups. This likely reflects the rising rates of schooling over the course of the twentieth century and well-documented trends in obesity rates more recently[26,39]. However, heart disease shows a steep decline in prevalence that is most likely due to age rather than cohort effects. Likewise, when we examine the variance over time of the phenotypes in Figure S5 (Panel B)we find patterns that match the mean phenotypic shifts across cohorts (with the exception of education, where the variance decreases in the younger birth cohorts likely due to a ceiling effect on number of years of schooling). To address the confounding of variance effects with levels changes, we also calculated the coefficient of relative variation over time (Figure S5, Panel C). Here we see that the changing variances appear to have been entirely an artifact of the shifting means across cohorts.

The same pattern obtains when we considerpolygenic scores(Figure S6). Here we are able to more directly assess evidence of mortality selection since the PGS score should not reflect environmental shifts and thus and differences should be entirely due to mortality differences (or shifts in the mean levels of PGS scores in the population due to differential fertility among their parents by PGS). The only score for which we observe a significant trend across birth cohorts is education, suggesting that those with lower scores die earlier, causing the earliest birth cohorts (i.e. the oldest respondents) to display significantly greater mean values than those with lower scores. It is interesting to note, then, that perhaps the education score is the better predictor of longevity even when compared to scores that are meant to directly capture health phenotypes. However, the variance in PGS, unlike that of phenotype, is relatively flat for all cases (Panel B of Figure S6), with the exception of height (which shows declining variance across cohorts, $p=0.0122$). The direction of change in the case of height PGS variance by birth cohort implies an underestimation of the trend we report. Namely, less variance in more recent cohorts would lead to attenuation bias due to decreased leverage for estimation in the younger groups, but what we observe is a larger effect in later cohorts despite such possible attenuation bias. The results of Panel B are largely unchanged when we consider the coefficient of variation for the scores (Panel C).

Even the existence of changing coefficients of variation, variance or means over time does not necessarily mean that our results are reflecting mortality bias. Assuming that the genetic effect is constant over time, what is the bias introduced by mortality selection on the estimated genetic effect? In earlier birth cohorts, those with advantageous genetics makeups are likely to be overrepresented relative to the population from those birth cohorts. This will cause a restriction of range such that the estimated genetic effect in earlier birth cohorts is biased towards zero. Thus, a constant genetic effect would not explain the education finding (since the effect in the earlier birth cohorts is larger) but may be related to the finding for height and BMI.

Another possibility for mortality bias to occur is in the relationship between PGS and its associated phenotype, is if there is differential mortality by the covariance of PGS and phenotype. If individuals with low values of the underlying "healthy" genotypes (as measured by the PGSs) and high levels of phenotypic outcomes (or the converse) died earlier, we might observe a stronger correlation between genotype and phenotype in the older cohorts because of differential mortality (or vice versa). This is highly unlikely. Let us take the example of education: A vast research literature suggests that education is positively related to longevity[40]. If the PGS exerts a similar positive effect on longevity (independent of education), then individuals with low educational attainment and low values of the PGS would face the highest mortality rates. In that case, selection bias would work to attenuate the relationship between the PGS and education. Since this selection bias would be greater for the older cohorts, it would cause us to *underestimate* the decline in the importance of genetic factors.

Another possibility is that the PGSs display non-linear effects and when people with low (or high) values on an attribute have an increased chance of early mortality, then they shift the remaining sample to a part of the distribution where the non-linearity evinces what appears to be a cohort change but is really just a local effect that is greater (or lesser) due to the truncation of the distribution. Since we have seen that mean values on the PGSs do change for some phenotypes, this could generate spurious cohort effects. We estimated regression equations where each phenotype was predicted by its PGS as well as a quadratic term in addition to the birth cohort variable. In most cases the quadratic term was not statistically significant (see Table S5, below). However, there were exceptions: Education showed a positive quadratic effect for its PGS as did the PGS for depression. (The BMI PGS was positive and just above conventional alpha levels.) For depression, this suggests that the lack of a trend in Figure 1 of the main text could be a combination of an upward trend in actual penetrance combined that is suppressed

by the fact that the mean depression PGS in the population has declined in more recent cohorts, moving the observed distribution of effects to a part of the curve where its impact is lessened. For education, the effect goes in the same direction as the observed change: the effect of the education PGS seems to decline in more recent cohorts; however, this could partially reflect the declining mean values of the education PGS in more recent cohorts, moving the observed effect to a lower part of the distribution where it is weaker. (Meanwhile there is not a significant change in the mean PGS for BMI across birth cohorts.) When we added a cubic term, it was insignificant for education and BMI, but negative for depression, suggesting a possibly more complicated relationship—i.e. gene-gene interactions or gene-environment interactions at work.

Gene-environment correlation and GxE parameter estimates

Some researchers have noted that gene-environment interaction (GxE) parameter estimates are sensitive to the presence gene-environment correlation (rGE).[42] This may be relevant to our inquiry because one can see some evidence rGE in Panel A of Supplementary Figure S6. Specifically, the average standardized PGS for education appears to decline across birth cohorts. While the correlation between PGS and cohort is very small in magnitude (r=-.04) we evaluated the sensitivity of our GxE parameter estimates to all possible values of rGE. We simulated 100,000 samples with values from a covariance matrix for PGS, education, and cohort that are similar to our empirical values. We randomly drew a correlation between PGS and cohort that ranged from -.90 to -.01 and estimated the gene-environment interaction model comparable to our own.

Figure S7 plots the absolute values of the test-statistics from each model. In total, 4,987 tests provided a significant interaction which is in line with the expected false-positive rate. The value of 1.96 is highlighted to illustrate the traditional level of significance ($p<.05$). As shown in this figure, there is no discernable pattern in the median test-statistic across the range of possible rGE values. The results from a linear model predicting the absolute value of the test statistic with cohort and the results of a logistic regression model predicting significance as a function of cohort both produced non-significant results. The presence of rGE may be important for the interpretation of GxE estimates in general, but given the empirical correlations between education and cohort and PGS and education that we observed, our results are robust to this form of rGE.

Discovery Sample Ascertainment Bias

It could theoretically be possible that any changes across cohorts in either the predictive accuracy of polygenic scores, are not the result of changes in the overall genetic component of the phenotypic variation (i.e. increasing or decreasing genetic effects or assortment) but rather the fact that those particular alleles that are associated with either phenotype or spousal genotype in one cohort are not predictive in another cohort (while alternative scores would be equally assortative or predictive). That is, it could be that any changes across birth cohorts merely reflect ascertainment bias due to the birth cohort distribution of the discovery samples. Namely, even if genetic factors as a whole are similarly important across birth cohorts, if *different* genetic factors matter for different birth cohorts, then a PGS constructed with weights from younger birth cohorts may have worse predictive power in older birth cohorts. Specifically, if the discovery analysis was done on younger birth cohorts, it would come as no surprise that the score predicts better for the younger group in the HRS (or vice versa). To find out whether this was driving the dynamics report here, we went back to the original studies that formed the bases of the meta-analyses of the consortia GWAS on which our polygenic scores are based. Information about the birth date distribution was not available for all of studies for all cohorts. For those that were available, we computed a weighted average of the birth year for the discovery sample of that particular polygenic score. These birth cohort means are reported in Tables S7-S11, below. The means summarized from that table are as follows:

1. GIANT (Height): 1943
2. GIANT (BMI): 1942
3. SSGAC (education): 1947
4. PSYCHE (depression): 1959
5. CARDIO (heart disease): 1949

In the case of all of the phenotypes, the mean year of birth was more recent than the median in our sample (1938). Thus, any ascertainment bias would work in the direction of finding greater predictive power in later cohorts. Taking the case of education, by way of example, the mean year of birth for all the cohorts in the discovery sample is 1947, whereas the mean year of birth for those included in our HRS analysis is 1938.Since our PGS is estimated on younger cohorts on average, then this source of bias would cause it to predict education better for the younger cohorts in the HRS. Thus, this source of bias is also unlikely to drive the pattern we show above.

As an additional exercise to insure we were not suffering from ascertainment bias as to the particular weighting of SNPs in the score calculations, we calculated additive heritability measures for young and old cohorts using a median birth year split (1938), deploying the GREML method as described elsewhere[41]. This approach does not rely on any discovery sample, but merely calculates the additive heritability based on the SNPs in the sample. When we compare younger and older birth cohorts in Figure S8, below, we do not find any striking patterns that would suggest systematic bias as compared to what we see in the reported in Figure 1 of the main text. (Though it must be said that GREML requires larger sample sizes than we obtain when we perform the median split; thus the error bars are quite wide for the h_{snps} estimates.) None of these median splits show statistically significant differences inheritability estimates nor any pattern that consistently contradicts what we report with respect to the PGS effects in Figure 1 of the main text.

Comparison with Twin-Based Estimates

Twin based models (as well as GREML models) could produce different results from the PGS analysis for several reasons. One could be that they pick up a larger portion (perhaps all) of the additive heritability while the measured PGS picks up only a portion of it. We tried to address this possibility by running GREML split by the median birth year as mentioned above. However, we were underpowered to detect any differences (see Figure S8). As also mentioned above, SIMEX analysis, which attempts to correct for the measurement error in the PGS failing to capture the entire additive heritability, does not change our results—in fact, this adjustment makes it stronger. So we are left to speculate as to why the results may be different between twin models and our approach.

In twin models, one cannot tell whether the change in additive heritability as a proportion of the total variance is due to the changing degree of environmental variation (C + E) or due to a change in the distribution of genetic variation. With the PGS we can measure this directly, as we do in Figure S6, Panel B, where it appears to be constant for the education PGS. We thus conclude that our observation is due to shifting environmental variation and not genetic variation. That said, this interpretation begs the question of why the PGS is not a random "subsample" of the additive genetic variation and/or calls into question the earlier twin methods. We suspect that it is due to problems with the external validity of twin samples and with the fact that many different samples are used across many populations, making

trends difficult to ascertain, at best. In fact, more recent, twin-based research shows that trends may be different for men and woman and overall are ambiguous at best[42].

The differences between the present study and Branigan et al. (2013)[20] are worth specifically noting. Branigan and coauthors performed a large-scale meta-analysis of twin-based heritability estimates of educational attainment. They found that—contrary to what we report here—younger cohorts demonstrated higher reported heritabilities. In addition to the fundamental differences between PGS and twin-based models as discussed above, there are a number of other differences in analysis strategy worth noting. First, there is little concordance in birth cohorts between their study and ours. In Branigan et al. the cohort split is dichotomous before and after the 1950 birth year. This split falls toward the end of our range of birth cohorts (1920-1955). Thus, it is entirely possible that during the early- to mid-20th Century, omnibus genetic effects on education declined before rising in the post-World War II period. Second, as an anonymous reviewer noted, "the heterogeneity across studies in Branigan et al. is quite significant with Cochran I2 $P < 0.05$. Thus, the discrepancy can be just viewed as the heterogeneity between the HRS study sample and the studies in Branigan et al." Lastly, this reviewer also suggested that differences could arise from gender dynamics: "The meta-analysis was adjusted for gender effect and the effect was quite significant for the heritability of education." It could be possible that gender moderates the penetrance of educational PGS by birth cohort. This possibility is made all the more salient given the rapid rise in women's education levels that occurred in the post-1950, pooled birth cohorts they studied. To test whether the trends in genetic penetrance differed by gender, we ran models for educational attainment (and the other phenotypes as well) that included—in addition to all the variables in our main models—a three-way interaction term between gender, PGS and birth cohort (as well as two-way interactions between gender and PGS, on the one hand, and birth cohort, on the other). These results are presented in Table S6, below. For none of the phenotypes is this interaction significant. This does not, of course, rule out the possibility that we are underpowered to detect such a "true" three-way interaction or that such an interaction is present when comparing later birth cohorts than we study (as did Branigan et al.).

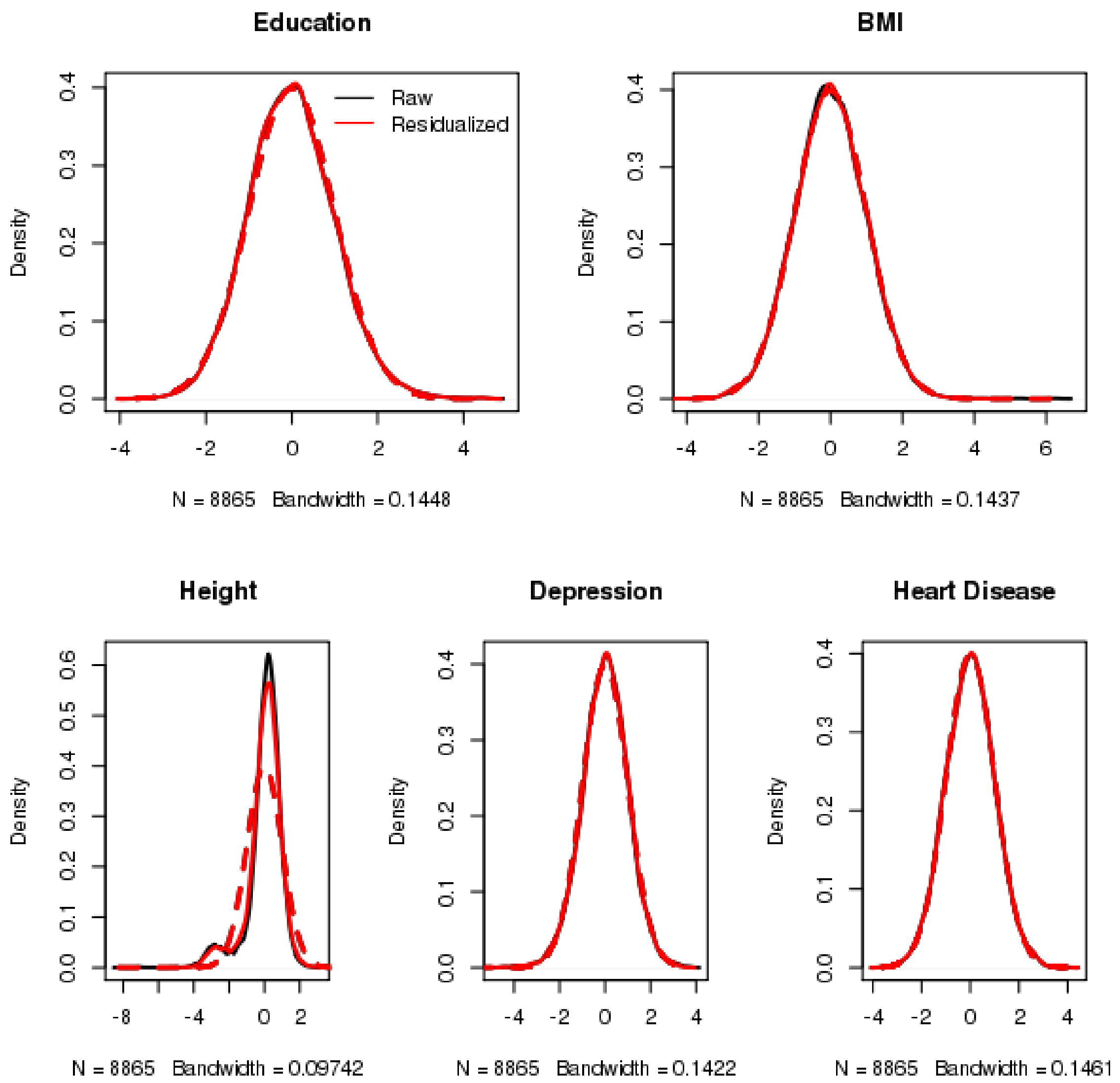

Figure S1: Densities before (black) and after (red) residualization by 10 PCs. Normal curves with the observed mean and SD are show in dashed lines.

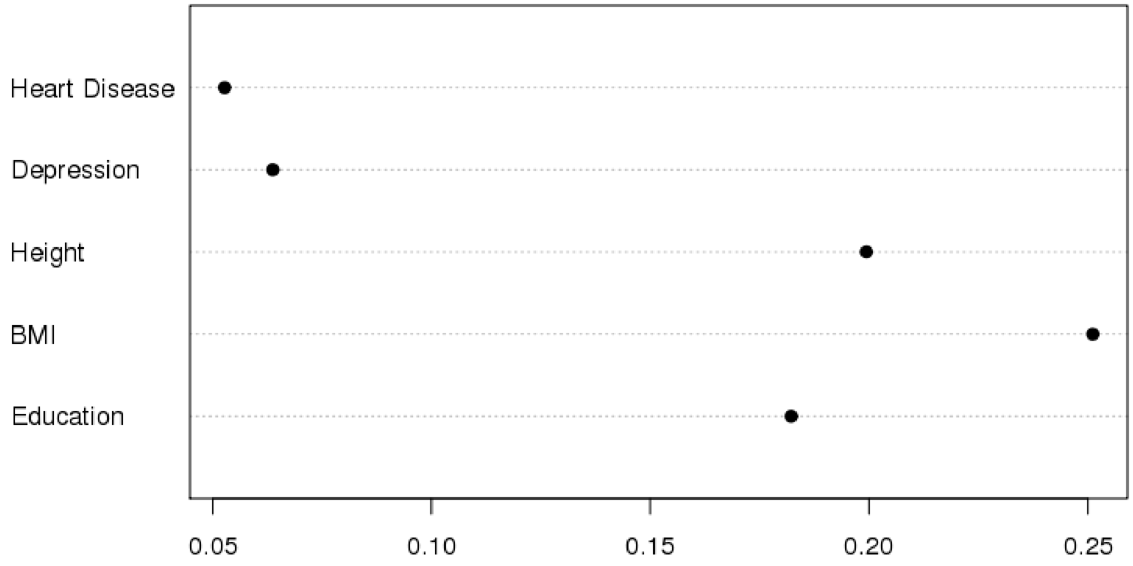

Figure S2: Graphical representation of correlations between polygenic scores and phenotypes.

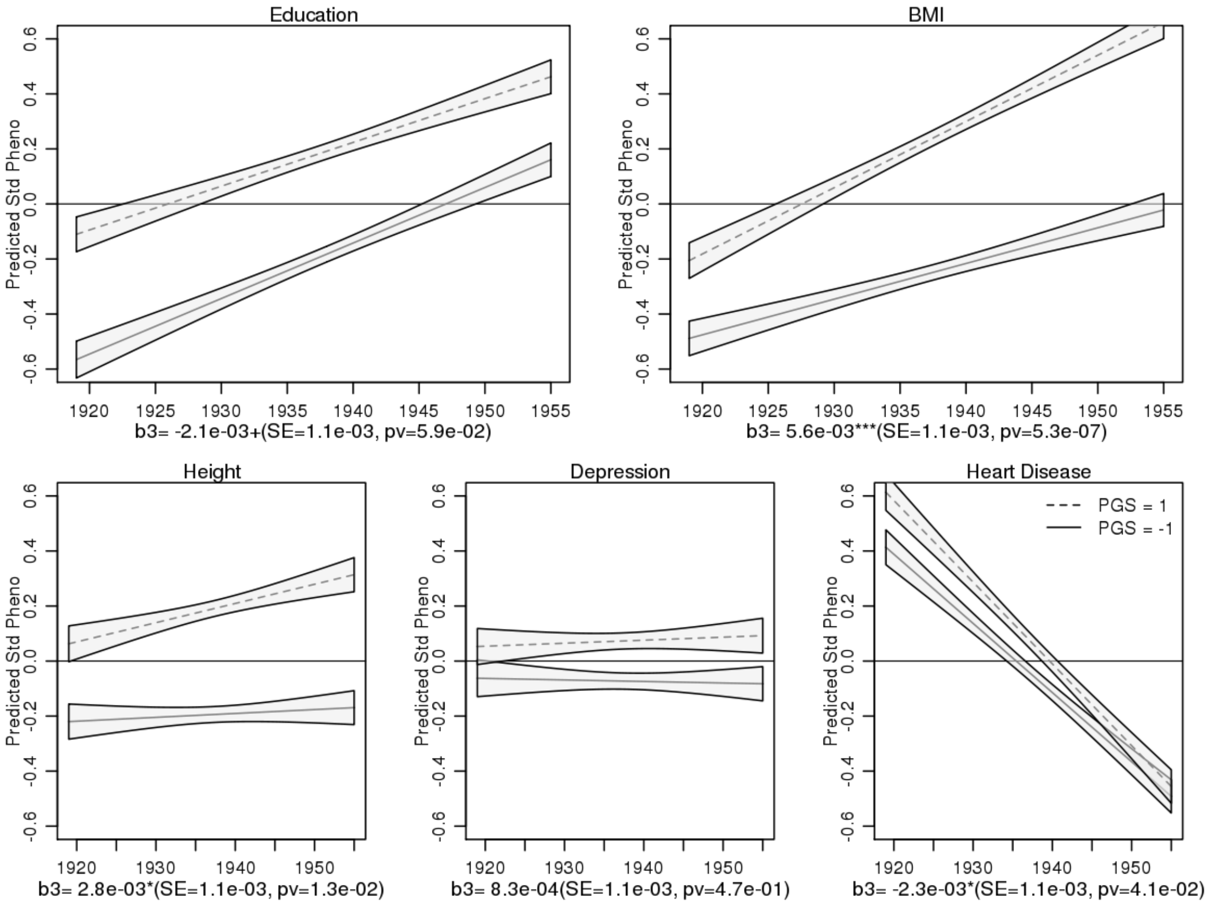

Figure S3: Version of Figure 1 from main text polygenic scores not residualized on top 10 PCs.

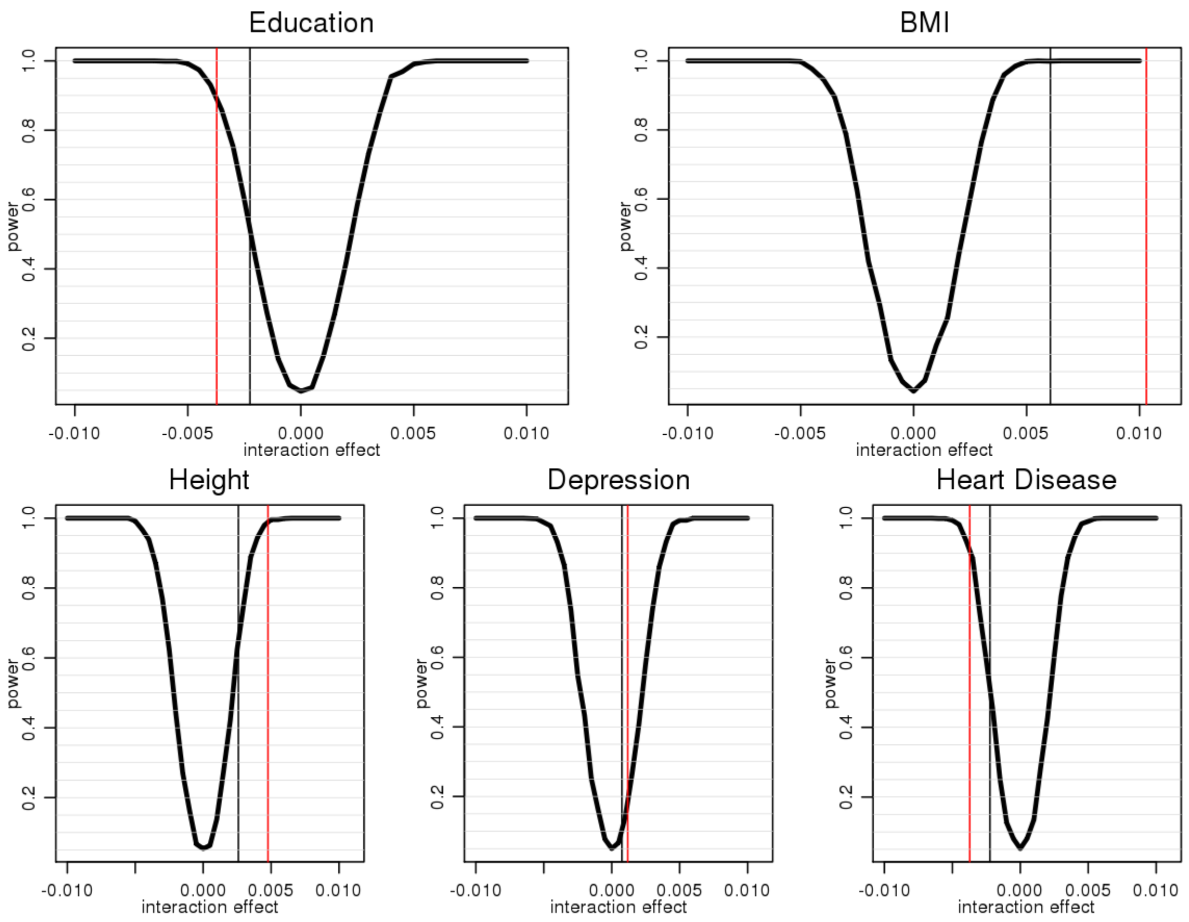

Figure S4: Results from power analysis over a range of potential interaction effects. Vertical black and red lines represent observed interaction effect and SIMEX estimated interaction respectively.

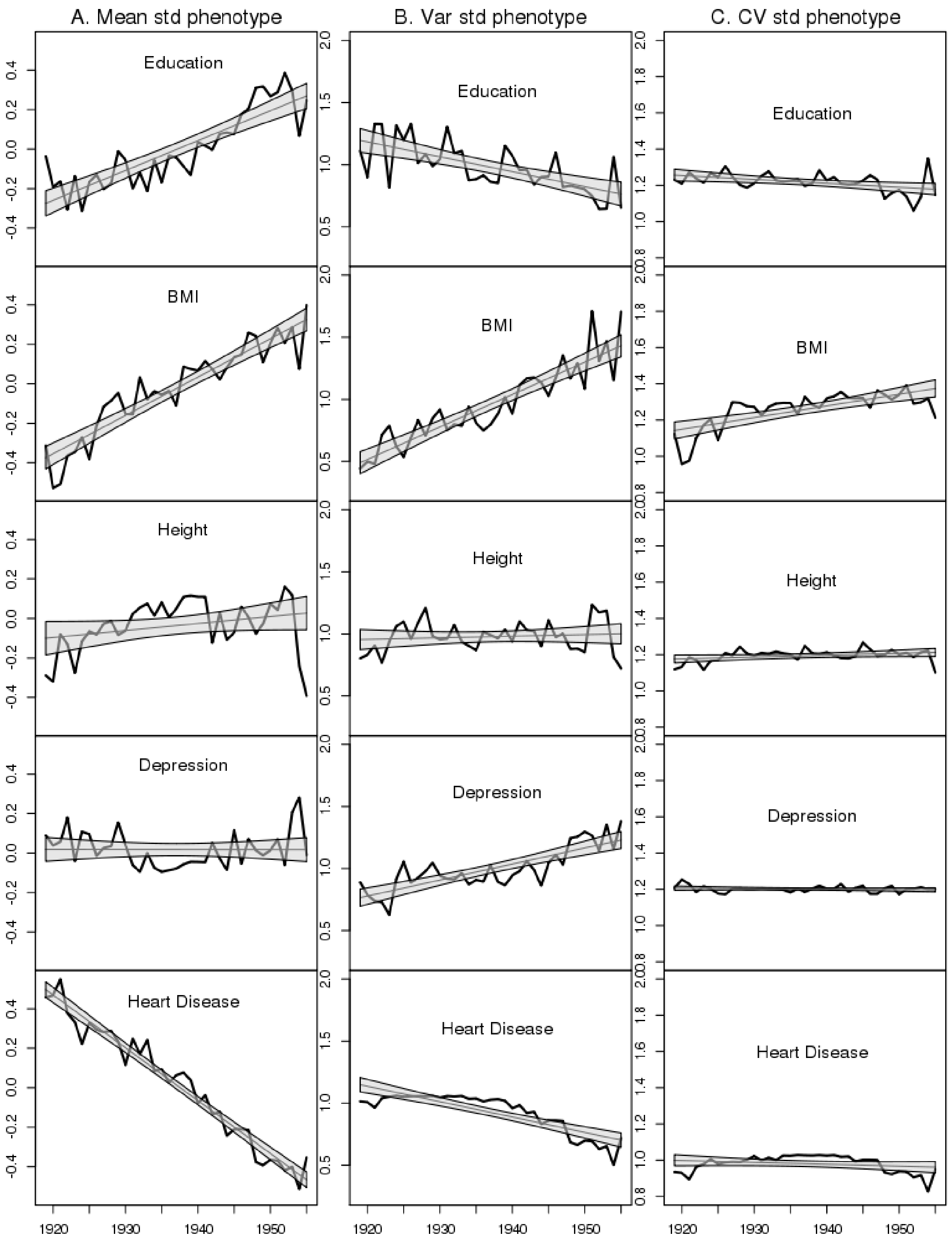

Figure S5: Mean standardized phenotypes (Panel A), variance in standardized phenotypes (Panel B) and coefficient of relative variation (standard deviation divided by mean) (Panel C), by HRS birth cohort.

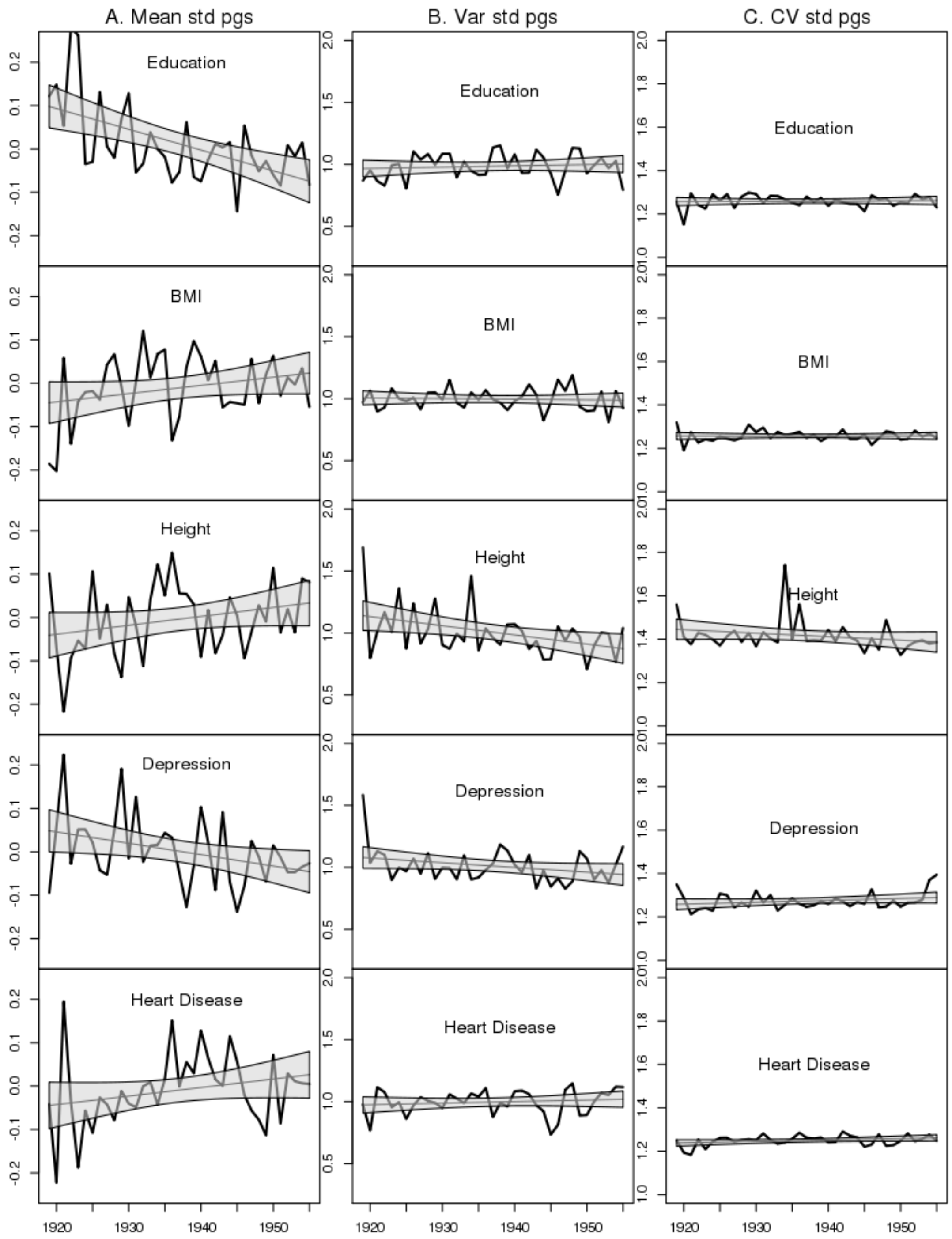

Figure S6: Mean standardized polygenic scores (Panel A) and variance in standardized polygenic scores (Panel B) by birth cohort in the HRS.

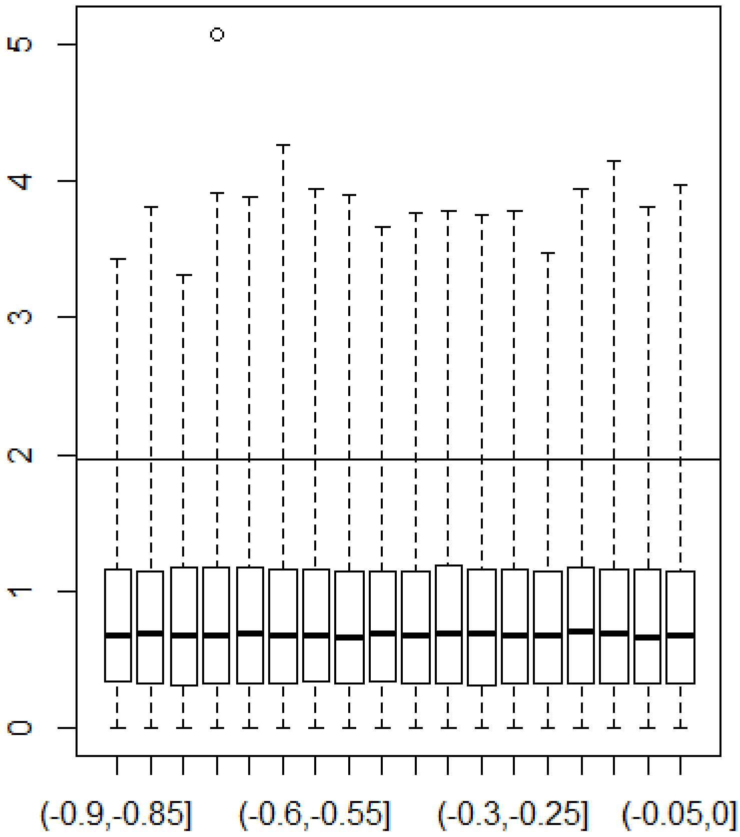

Figure S7. Distribution of GxE test statistics as a function of rGE levels.

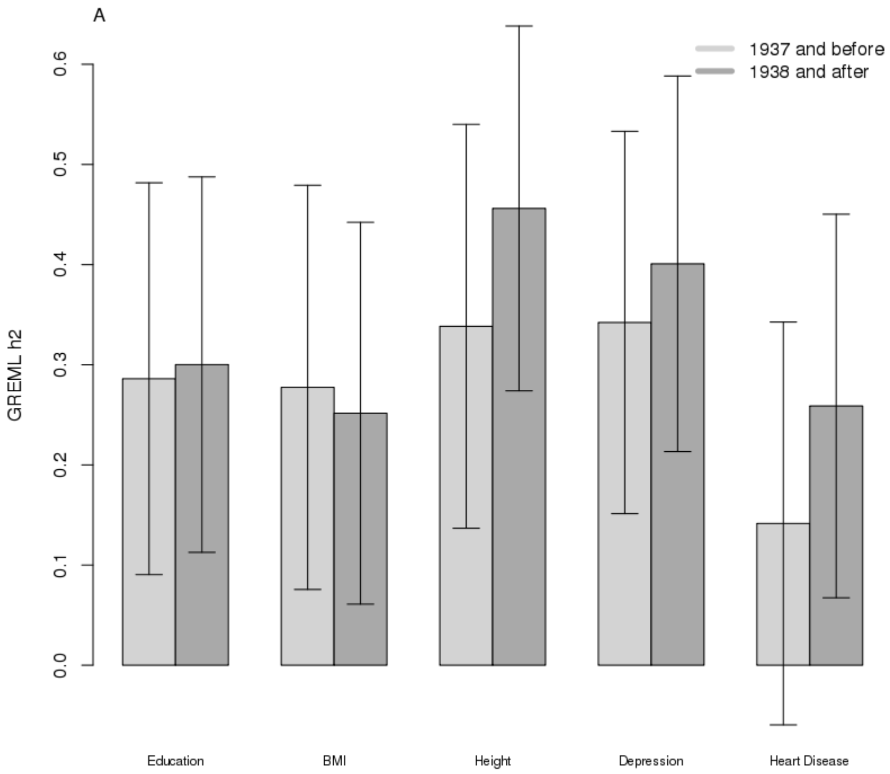

Figure S8: GREML estimates of h_{snps}, by birth cohort in the HRS for selected phenotypes.

Table S1: Sample means and standard deviations for HRS genotyped respondents in analytic sample (non-Hispanic Whites born between 1919 and 1955).

	Mean-all	SD-all
Birth Year	1938	9
Female	0.57	0.5
Education	13.2	2.6
BMI	27.5	5.05
Height	1.7	0.1
Heart Dis.	0.39	0.49
CES-D	1.21	1.33
N	8865	

Table S2: Correlation of phenotype & polygenic scores for all non-Hispanic white genotyped respondents (N=8,865).

	Correlation	CI		R^2 (GxE)	Power % (K=3, α=.05)	Partial R^2 (GxE term)	Power % (K=1, α=.05)
Education	0.182	0.162	0.202	.061	100	.0004	48
Height	0.199	0.179	0.219	.042	100	.0006	65
BMI	0.251	0.232	0.271	.095	100	.0030	100
Depression	0.064	0.043	0.084	.004	100	.0005	57
Heart Dis.	0.053	0.032	0.074	.066	100	.0004	48

Table S3. Regression estimates underlying Figure 1 and SIMEX adjusted estimates

	Main Analyses (Figure 1 of main text)				
	Education	Height	BMI	Depression	Heart Disease
pgs	4.550*	-4.817*	-11.474***	-1.386	4.384*
	(2.195)	(2.176)	(2.146)	(2.214)	(2.166)
t	.018***	.004***	.019***	<.001	-.027***
	(.001)	(.001)	(.001)	(.001)	(.001)
pgs: t	-.002*	.003*	.006***	<.001	-.002*
	(.001)	(.001)	(.001)	(.001)	(.001)
Intercept	-34.954***	-7.864***	-36.077***	-.049	53.122***
	(2.174)	(2.191)	(2.129)	(2.233)	(2.163)
	SIMEX Adjusted Analyses				
	Education	Height	BMI	Depression	Heart Disease
pgs	7.528*	-8.921**	-19.559***	-2.178	7.302*
	(3.236)	(3.065)	(3.231)	(3.205)	(3.144)
t	.019***	.004***	.019***	<.001	-.028***
	(.001)	(.001)	(.001)	(.001)	(.001)
pgs: t	-.004*	.005**	.010***	.001	-.004*
	(.002)	(.002)	(.002)	(.002)	(.002)
Intercept	-36.118***	-7.500***	-35.952***	-.699	53.341***
	(2.173)	(2.178)	(2.124)	(2.234)	(2.164)

Standard errors in parentheses.
*** $p < .001$; ** $p < .01$; * $p < .05$

Table S4: Comparison of standard and Huber-White Corrected regression results (focusing on interaction estimates).

	Education	Height	BMI	Depression	Heart Disease
Standard	-.002*	.003*	.006***	<.001	-.002*
	(.001)	(.001)	(.001)	(.001)	(.001)
HW-	-.002*	.003*	.006***	<.001	-.002*
Corrected	(.001)	(.001)	(.001)	(.001)	(.001)
N	8851	8865	8862	8865	8865

Standard errors in parentheses.
*** $p< .001$; ** $p < .01$; * $p < .05$

Table S5: Testing for non-linearities in effect of PGSs on phenotypes

	Education	Height	BMI	Depression	Heart Disease
pgs	.185***	.200***	.249***	.067***	.057***
	(.010)	(.011)	(.010)	(.011)	(.010)
pgs^2	.018**	.003	.013	.015*	<.001
	(.007)	(.003)	(.007)	(.007)	(.007)
t	.018***	.004***	.019***	<.001	-.027***
	(.001)	(.001)	(.001)	(.001)	(.001)
Intercept	-34.789***	-7.826***	-35.853***	-.615	52.987***
	(2.172)	(2.191)	(2.132)	(2.233)	(2.162)

Standard errors in parentheses.
*** $p < .001$; ** $p < .01$; * $p < .05$

Table S6: Models with Gender Interaction Effects (Standard Errors in Parentheses)

	Education	BMI	Height	Depression	Heart Disease
PGS	11.244	-10.500	-2.260	-5.994	.464
	(7.330)	(7.262)	(4.823)	(7.542)	(7.206)
BYear	.018***	.014***	.004†	.008*	-.042***
	(.004)	(.004)	(.002)	(.004)	(.004)
Gender	-.540	-5.810	-3.253	10.075*	-18.100***
(Fem=1)	(4.406)	(4.315)	(2.887)	(4.490)	(4.378)
PGS x	-.006	.006	.001	.003	<.001
BYear	(.004)	(.004)	(.002)	(.004)	(.004)
PGS x	-4.249	-.556	-.216	3.188	2.710
Gender	(4.433)	(4.359)	(2.884)	(4.487)	(4.362)
BYear x	<.001	.003	.001	-.005*	.009***
Gender	(.002)	(.002)	(.001)	(.002)	(.002)
PGS x	.002	<.001	<.001	-.002	-.001
BYear x	(.002)	(.002)	(.001)	(.002)	(.002)
Gender					
Intercept	-34.431***	-27.200***	-5.862	-15.829*	81.400***
	(7.330)	(7.174)	(4.801)	(7.469)	(7.287)
N	8851	8862	8865	8865	8865

Table S7: GIANT—Height[6]

PERCENT TOTAL SAMPLE, BIRTHYEAR ASCERTAINED: 63.5%
WEIGHTED AVERAGE BIRTHYEAR: 1943.012

Study	Year(s)	Ages [mean,sd]	Birth Cohort	*N*
The AIDS Clinical Trials Group (ACTG)			NA	1055
Athero-Express Biobank Study (AE)			Mean: 1934	686
Anglo-Scandinavian Cardiac Outcome Trial (ASCOT)			Mean: 1935	3802
Baltimore Longitudinal Study on Aging (BLSA)			Mean: 1933.9	844
B-Vitamins for the Prevention of Osteoporotic Fractures (B-PROOF)			NA	2669
Data from an Epidemiological Study on the Insulin Resistance Syndrome (DESIR)			NA	716
Danish National Birth Cohort-Preterm Delivery Study (DNBC)			Mean: 1966.69	1802
Estonian Genome Center, University of Tartu (EGCUT-370)			NA	866
Estonian Genome Center, University of Tartu (EGCUT-OMNI)			NA	1356
Erasmus Rucphen Family Study (ERF)			NA	2726

Family Heart Study (FamHS)	Mean: 1942 (Males); 1936 (Females)	1463
Health, Aging, and Body Composition Study (Health ABC)	Mean: 1925	1655
Health, Risk Factors, Training and Genetics (HERITAGE) Family Study	Mean: 1957	500
HYPERGENES (Cases)	NA	1900
HYPERGENES (Control)	NA	1841
Invecchiare in Chianti (InCHIANTI)	Mean: 1928	1138
Charles Bronfman Institute for Personalized Medicine BioMeBioBank Program (IPM)	Weighted Average: 1946.596	2867
Lifelines Cohort Study (LifeLines)	Mean: 1959.97	8118
Leiden Longevity Study (LLS)	Mean: 1942	1903
London Life Sciences Prospective Population Study (LOLIPOP-EW610)	NA	927
London Life Sciences Prospective Population Study (LOLIPOP-EWA)	NA	513
London Life Sciences Prospective Population Study (LOLIPOP-EWP)	NA	651

Dutch and Belgian Lung Cancer Screening Trial (NELSON)	Mean: 1945	2668
Prostate, Lung, Colorectal, and Ovarian Cancer Screening Trial (PLCO2 Controls)	NA	1193
PLOC2 (Cases)	NA	2976
Prevention of REnal and Vascular ENdstate Disease (PREVEND) Study		3624
Precocious Coronary Artery Disease (PROCARDIS)	Mean: 1945	7000
The PROspective study of Prevastatin in the Elderly at Risk for Vascular Disease (PROSPER/PHASE)	NA	5244
Quebec Family Study (QFS)	NA	860
Twin Study at Queensland Institute of Medical Research (QIMR)	Mean: 1951	3627
Relationship between Insulin Sensitivity and Cardiovascular Disease Study (RISC)	Mean: 1959	1031
Study of Health in Pomerania (SHIP-TREND)	Mean: 1945	986
Tracking Adolescents' Individual Lives Survey (TRAILS)	Mean: 1971	1139
TWINGENE	Mean: 1922	9380

Table S8: GIANT—BMI[7]

PERCENT TOTAL SAMPLE, BIRTHYEAR ASCERTAINED: 82.91%
WEIGHTED AVERAGE BIRTHYEAR: 1942.363

Study	Year(s)	Ages [mean,sd]	Birth Cohort	*N*
Previous GWAS Studies (Speliotes)				
Age, Gene/Environment Susceptibility-Reykjavik Study (AGES)	Baseline 2002	34-77 [m-49.69, f-52]	1925-1968 Mean: 1952 (Males) 1950 (Females)	3207
Amish Hereditary and Phenotype Heart Study (Amish HAPI Heart Study)	Baseline 2002	20-98 [m-45.90, f-47.50]	1904-1982 Mean: 1956 (Males); 1954 (Females)	905
Atherosclerosis Risk in Communities Study (ARIC)	Baseline 1987-1989	44-66 [m-54.69, f-53.97]	1921-1945 Mean: 1934	8108
British 1958 Birth Cohort--Type I Diabetes Genetic Consortium Controls (B58C-T1DGC)	Baseline 1958	44.5-46	1958 only	2587
British 1958 Birth Cohort--Wellcome Trust Case Control Consortium Controls (B58C-WTCCC)	Baseline 1958	44.5-46	1958 only	1479
The British Genetics of Hypertension (BRIGHT) Study (BRIGHT-WTCCC-HT)	2002 (hypertension module baseline)	21-85 [m-56.29, f-57.43]	1917-1981 Mean: 1945	1960
WTCCC Coronary Heart Disease Cases (CAD_WTCCC)	2005	35-82 [m-59.97, f-60.30]	1923-1970 Mean: 1945	1876
Cancer Prostate in Sweden 1 (CAPS1)	Baseline 2001-2003	44.90-81.10 [67]	1920-1962 Mean: 1935	1011
Cancer Prostate in Sweden 2 (CAPS2)	Baseline 2001-2003	44.90-82.20 [66]	1919-1962 Mean: 1936	2002
Cardiovascular Health Study (CHS)	Baseline June 12, 1989	65-98 [m-73, f-71.90]	1891-1923/24 Mean: 1916 (Males); 1917 (Females)	3238

CohorteLausannoise (CoLaus)	2003	35-75 [m-52.92, f-53.88]	1928-1968<br>Mean: 1950	5409
Diabetes Epidemiology: Collaborative Analysis of Diagnostic Criteria in Europe (deCODE)	1997 Baseline	11.50-108 [m-64.74, f-57.94]	1889-1985<br>Mean: 1932 (Males); 1939 (Females)	26799
Diabetes Genetics Initiative of Broad Institute and MIT, Lund, Novartis (DGI)	ND/NA	35-95 [63 case, 58 control]	NA<br>Mean: No baseline established	2405
Estonian Genome Center, University of Tartu (EGCUT)	2001 baseline (to 2007, rolling? not defined)	18-92 [m-40.62, f-42.88]	1909-1983<br>Mean: 1960 (Males); 1958 (Females)	1417
European Prospective Investigation into Cancer and Nutrition-Obesity Study (EPIC-Obesity Study)	Rolling recruitment, 1993-1998	45-74 [59]	1919-1953<br>Mean: Approx. 1937	2415
Erasmus Rucphen Family-EUROSPAN (ERF)	Baseline 2002 (-2005)	18-92.10 [50]	1910-1987<br>Mean: 1953/1954	2060
Fenland Study (Fenland)	Active	30-62 at recruitment (30-57 in sample) [45]	1950-1975<br>Mean: Wave not specified	1402
Framingham Heart Study (FRAM)	Baseline 1948; 2002-2005 third.	21-72 [38]	(Assuming third cohort, 1930-1984)<br>Mean: 1964 approx.	8094
Finnish Twin Cohort (FTC)	Baseline 1975	26-76 (in meta) [64]	Approx. 1933-1983<br>Mean: Wave not determined	125
Finland-United States Investigation of NIDDM Genetics (FUSION)	NA	40-83 [62]	NA<br>Mean: Wave not determined	1092
Health 2000/GENMETS Substudy (GENMETS)	2000/2001	30-75	1925-1971<br>Mean: 1948 (Female cases); 1950 (Other)	1681

German Myocardial Infarction Family Study I (GerMiFSI)	1997-2002	32-82	1915-1970<br>Mean: Approx.<br>Mean: 1943 (Males); 1940 (Females)	600
German Myocardial Infarction Family Study II (GerMiFSII)	1997-2002	29-90	1907-1973<br>Mean: 1940 (Males); 1937 (Females)	1124
Cooperative Health Research in the Region Augsburg, KOoperativeGesundheitsforschung in der Region Augsburg (KORA3)	1994-1995	25-74 (25-69 in sample)	1925-1970<br>Mean: 1941	1644
Cooperative Health Research in the Region Augsburg, KOoperativeGesundheitsforschung in der Region Augsburg (KORA4)	1999-2001	25-74	1925-1976<br>Mean: 1946	1814
MICROS-EUROSPAN (MICROS)	2002-2003	18-88	1914-1985<br>Mean: 1957	1097
Myocardial Infarction Genetics Consortium (Migen)	Baseline 1997	<51 (male), <61 (female);<br><45 (Italian subsample, both sexes)<br>*[48.8, 8.2]*	1937-undefined<br>Mean: 1948 (MGH sub)	2681
WTCCC National Blood Service Donors (NBS_WTCCC)	ND	42-69	NA<br>Mean: ND	2681
Northern Finland Birth Cohorts (1966)--(NFBC-1966)	Initial collection 1969 for '66 cohort	All 31	1966 Only	4497
The Nurse's Health Study (NHS)	1976 baseline	30-55 first NHS cohort<br>(39-66 in sample)	1921-1946<br>Mean: 1932 (based on second wave)	2265
Northern Sweden Population Health Study-EUROSPAN (NSPHS)	2006	14-91	1915-1992<br>Mean: 1959	656
Netherlands Twin Register & the Netherlands Study of Depression and Anxiety (NTRNESDA)	NESDA baseline 1996<br>NTR 1991	18-81	1910-1978<br>Mean: Can't determine, pooled data	3516

Orkney Complex Disease Study-EUROSPAN (ORCADES)	Baseline enrollment 2005	17-97	1908-1988<br>Mean: 1951/1952 (case, control)	716
Prostate, Lung, Colorectal, Population-Based and Ovarian Cancer Screening Trial (PLCO)	Pilot 1994, recruitment to 2000	55-74	1920-1945<br>Mean: 1933	2238
Rotterdam Study 1 (RS-1)	Baseline 1990	55-99 [69]	1891-1935<br>Mean: 1921	5974
Nijmegen Bladder Cancer Study and Nijmegen Biomedical Study (RUNMC)	2002-2003 baseline	24-91	1911-1979<br>Mean: 1939 (Males); 1947 (Females)	2873
Swedish and Singapore Breast Association Consortium (SASBAC)	Sw: 1993-1995	Sw: 50-74 (63-75 in sample) [62]	1918-1932<br>Mean: 1932 (in meta analysis)	1559
Studies of Epidemiology and Risk Factors in Cancer Heredity/UK Ovarian Cancer Population Study (SEARCH/UKOPS)	2001-2005	20-91	1910-1985 [57]<br>Mean: 1946	1710
Study of Health in Pomerania (SHIP)	1996	20-79 (20-81 in sample)	1915-1976 [48]<br>Mean: 1948	2073
WTCCC Type 2 Diabetes (T2D-WTCCC)	1996	29-96	1900-1967<br>Mean: 1938	798
TwinsUK (TwinsUK)	1993	16-85 (16-76 in sample)	1917-1977<br>Mean: 1947	1479
VIS EUROSPAN and KORCULA (VIS)	2007 (Korcula)	18-93	1914-1989<br>Mean: 1951 (Korcula)	795
New or Updated GWAS Studies				
Athero-Express Biobank Study (AE)			Mean: 1934	622

Anglo-Scandinavian Cardiac Outcome Trial (ASCOT)	Mean: 1935	3802
Baltimore Longitudinal Study on Aging (BLSA)	Mean: 1933.9	844
Busselton Health Study (BSN-BHS)	Mean: 1919	1327
Genetic Predisposition of Coronary Heart Disease in Patients Verified with Coronary Angiogram (COROGENE)	NA	3756
Data from an Epidemiological Study on the Insulin Resistance Syndrome (DESIR)	NA	716
Danish National Birth Cohort-Preterm Delivery Study (DNBC)	Mean: 1966.69	1802
Estonian Genome Center, University of Tartu (EGCUT-370)	NA	866
Estonian Genome Center, University of Tartu (EGCUT-OMNI)	NA	1356
Erasmus Rucphen Family Study (ERF)	NA	2726
Family Heart Study (FHS)	Mean: 1942 (Males); 1936 (Females)	1463
Finnish Genetic Study of Arrhythmic Events (FinGesture)	Mean: 1939.81 (Males); 1933.56 (Females)	943
Gothenburg Osteoporosis and Obesity Determinants Study (GOOD)	NA	938

Health, Aging, and Body Composition Study (Health ABC)	Mean: 1925	1655
Helsinki Birth Cohort Study (HBCS)	Mean: 1940.85	1726
Health, Risk Factors, Training and Genetics (HERITAGE) Family Study	Mean: 1957	500
Invecchiare in Chianti (InCHIANTI)	Mean: 1928	1139
Charles Bronfman Institute for Personalized Medicine BioMeBioBank (IPM)	Mean varies between subsamples--1939-1954	2867
Lifelines Cohort Study	Mean: 1959.97	8118
Leiden Longevity Study (LLS)	Mean: 1942	1903
London Life Sciences Prospective Population Study (LOLIPOP-EW610)	NA	927
London Life Sciences Prospective Population Study (LOLIPOP-EWA)	NA	513
London Life Sciences Prospective Population Study (LOLIPOP-EWP)	NA	651
Molecular Genetics of Schizophrenia/NIMH Repository Control Sample (MGS)	NA	2597
Dutch and Belgian Lung Cancer Screening Trial (NELSON)	Mean: 1945	1135

Prostate, Lung, Colorectal, and Ovarian Cancer Screening Trial (PLCO2)	NA	4175
Precocious Coronary Artery Disease (PROCARDIS)	Mean: 1945	7279
The PROspective Study of Pevastatin in the Elderly at Risk for Vascular Disease (PROSPER/PHASE)	NA	5244
Quebec Family Study (QFS)	NA	860
Twin Study at Queensland Institute of Medical Research (QIMR)	Mean: 1951	3627
Relationship between Insulin Sensitivity and Cardiovascular Disease Study (RISC)	Mean: 1959	1031
Rotterdam Study III (RSIII)	Mean: 1950	2006
Study of Health in Pomerania-TREND (SHIP-TREND)	Mean: 1945	986
Self-contained population from East Germany, European Descent (Sorbs)	NA	907
Tracking Adolescents' Individual Lives Survey (TRAILS)	Mean: 1971	1141
TWINGENE	Mean: 1922	9176
TwinsUK	Mean: 1947	3003

Women's Genome Health Study (WGHS)	Mean: 1939	22888
Cardiovascular Risk in Young Finns Study (YFS)	Mean: 1969	1989

Table S9: Social Science Genetics Association Consortium (SSGAC), Education[9]

PERCENT TOTAL SAMPLE, BIRTHYEAR ASCERTAINED: 100%
WEIGHTED AVERAGE BIRTHYEAR: 1947.271

Study	Year(s)	Ages [mean,sd]	Birth Cohort	N
Age, Gene/Environment Susceptibility-Reykjavik Study (AGES)	Baseline 2002	66-95 [76.41, 5.45]	1908-1936<br>Mean: 1926	3,212
Avon Longitudinal Study of Parents and Children (ALSPAC)		15-44 [28.69, 4.66]	1948-1977<br>Mean: 1962.95	6,919
Austrian Stroke Prevention Study (ASPS)		46-85 [65.51, 7.98]	1909-1949<br>Mean: 1931.97	848
Baltimore Longitudinal Study of Aging (BLSA)		30-101 [71.63, 15.55]	1902-1977<br>Mean: 1933.9	821
Cancer Hormone Replacement Epidemiology in Sweden (CAHRES)		66-92 [79, 6.2]	1919-1944<br>Mean: 1931	1,497
Cancer Prostate Sweden (CAPS)		49-81 [68, 7.5]	1921-1954<br>Mean: 1933 (Cases); 1935 (Controls)	459
Cleveland Clinic Foundation (CCF)		30-84 [59.39, 9.82]	1923-1978<br>Mean: 1947	485
CohorteLausannoise (CoLaus)	2003	34-75 [53.43, 10.75]	1928-1970<br>Mean: 1951	5410
Croatia Korcula (CRKOR)		30-98	1909-1979<br>Mean: 1949 (Korcula); 1955.9 (Split); 1944.8	2,124

			(Vis)	
Estonian Genome Center, University of Tartu (EGCUT)	2001 baseline (to 2007, rolling? not defined)	30-103	1905-1979<br>Mean: 1949	1,537
Erasmus Rucphen Family-EUROSPAN (ERF)	Baseline 2002 (-2005)	30-89 [51.77, 12.29]	1914-1974<br>Mean: 1951	2,380
Finnish FINRISK (FINRISK)		30-74 [46.28, 11.40]	1923-1977<br>Mean: 1946	1,823
Finnish Twin Cohort (FTC)		41-67 [54.88, 4.48]	1937-1961<br>Mean: 1948	729
Genetic Association Information Network Schizophrenia Controls (GAIN)		30-90 [55.48, 14.23]	1916-1976<br>Mean: 1950	1,164
Genetic Epidemiology Network of Arteriopathy (GENOA)		24-89 [55.33, 10.82]	1908-1974<br>Mean: 1942	1,439
Health ABC (HABC)		69-80 [73.77, 2.84]	1917-1928<br>Mean: 1923	1,659
Helsinki Birth Cohort Study (HBCS)		56-69 [61.47, 2.92]	1934-1944<br>Mean: 1940.85	1,717
Invecchiare in Chianti (InCHIANTI)		30-102 [69.95, 13.29]	1896-1970<br>Mean: 1928	1,164
KooperativeGesund heitsforschung in der Region Augsburg (KORA3)		30-69 [53.28, 9.20]	1925-1964<br>Mean: 1940	1,595

KooperativeGesundheitsforschung in der Region Augsburg (KORA4)	31-74 [53.93, 8.82]	1926-1969<br>Mean: 1946	1,809
Lifelines Cohort Studies (LIFELINE)	30-89 [48.57, 10.31]	1920-1980<br>Mean: 1959.97	7,493
Lothian Birth Cohort 1921 (LBC1921)	77-80	1921 Only	515
Lothian Birth Cohort 1936 (LBC1936)	67-71	1936 Only	1,003
Mother and Child Cohort of NIPH (MoBa)	20-34	1966-1976<br>Mean: 1971	759
Netherlands Study of Depression and Anxiety (NESDA)	30-65 [46.69, 9.35]	1939-1976<br>Mean: 1959	1,517
Northern Finland Birth Cohort 1966 (NFBC1966)	31	1966 Only	5,371
Non-Genetic Association Information Network Schizophrenia (nonGAIN)	30-90 [53.02, 13.88]	1916-1976<br>Mean: 1953	1,109
Netherlands Twin Register (NTR)	30-91 [51.39, 12.49]	1917-1980<br>Mean: 1954	2,650
Queensland Institute of Medical Research (QIMR)	30-101 [44.95, 10.09]	1900-1975<br>Mean: 1951	7,985
Rotterdam Study Baseline (RSI)	55-99 [69.18, 8.95]	1893-1938<br>Mean: 1922	5,806

Rotterdam Study Extension (RSII)	55-95 [64.97, 8.15]	1906-1944<br>Mean: 1935	1,641
Rotterdam Study Young (RSIII)	45-97 [56.11, 5.84]	1910-1960<br>Mean: 1950	2,014
Rush University Medical Center-Memory and Aging Project (RUSH-MAP)	55-101 [81.10, 6.66]	1901-1948<br>Mean: 1921	888
Rush University Medical Center-Religious Orders Study (RUSH-ROS)	60-102 [75.70, 7.35]	1896-1946<br>Mean: 1921	810
Study of Addiction: Genetics and Environment (SAGE)	30-65 [38.70, 5.68]	1938-1975<br>Mean: 1965	1,321
SardiNIA Study of Aging (SardiNIA)	30-101 [52.81, 14.25]	1900-1980<br>Mean: 1954	3,639
Study of Health in Pomerania (SHIP)	30-81 [53.27, 14.08]	1918-1971<br>Mean: 1945	3,556
Swedish Twin Register (STR)	47-89 [63.82, 8.74]	1916-1958<br>Mean: 1941	9,553
UK Adult Twin Registry (TwinsUK)	30-80 [51.03, 10.72]	1919-1978<br>Mean: 1949	2,619
Cardiovascular Risk in Young Finns Study (YFS)	30-45 [37.72, 5.01]	1962-1977<br>Mean: 1969	2,029

Table S10: Birth cohorts for discovery samples--Psychiatric GWAS Consortium, depression and mental health[8]

PERCENT TOTAL SAMPLE, BIRTHYEAR ASCERTAINED: 43.9%
WEIGHTED AVERAGE BIRTHYEAR: 1958.596

Segment from: (MDD) Major Depressive Disorder Working Group of the Psychiatric GWAS Consortium[44]

Study	Year(s)	Ages [mean,sd]	Birth Cohort	N
Bonn/Mannheim MDD Study	NA	47.5, 13.9	NA	
GAIN	2004-2007	18-65	1939-1989	NA
		[Cases: 42.6, 12.6; Controls: 45.1, 14.1]	Mean: 1963 (Cases); 1961 (Controls)	
The Genetics of Recurrent Early-Onset Depression (GenRED)	Genotyped in 2007/2008.	Cases=40.5, 11.9; Controls=52.5, 17.2	NA<br>Mean: 1967 (Cases); 1955 (Controls)	
Glaxo-Smith-Kline (GSK)	Sample II: 2003	SII: 35-75 years	SII only: 1928-1968	SII: 1544
			Mean: No descriptives	
GSK (Descriptives based on Genome-Wide Association and Meta Analysis of Bipolar Disorder in Individuals of European Ancestry)	NA	18-84 [47.1, 12.2]	1919-1985<br>Mean: 1956 (2003)	899
MDD2000/QIMR			1900-1978 (except UoE subsample--University of Edinburgh MDD)	
			Mean: NA	
Max Planck Institute of Psychiatry (MPI-P)	Multiple studies, no detailed information on cohorts/age ranges		Mean: NA	
RADIANT--Genetic-Based	~2005-2008	19-72	~1933 -1989	811

Therapeutic Drugs for Depression (GENDEP)			Mean: NA	
RADIANT--Depression Case Control Study (DeCC)	NA	18+ [Men: 48.59, 11.71; Female: 46.49, 12.31]	ND<br>Mean: NA	1237
The Depression Network Study (DeNT)	NA	18+ [Avgs: 42.39-51.67 through eight study locations]	ND<br>Mean: NA	
Sequenced Treatment Alternatives to Relieve Depression (STAR*D)	2001-2006	18-75	1926-1988<br>Mean: No Descriptives	4041
Children's Hospital of Philadelphia (CHOP)	NA	6-18	ND	2026
International Multisite ADHD Genetics Project Phase I (IMAGE)	2003-2007	5-17	1986-2002<br>Mean: No descriptives	
IMAGE II	2007	5-17	1986-2002<br>Mean: No descriptives	
Pfizer, Washington University and Mass General (PUWMa)	MGH: NA<br>WASHU: 1996-2002<br>UCLA: NA	6-17	WASH: 1979-1996<br>Mean: No descriptives	
Bipolar Study, University of Bonn and CIMH Mannheim (BOMA)	NA	NA	ND<br>Mean: No descriptives	675 (case)
Genetic Association Information Network (GAIN)/Bipolar Genome	NA	NA	ND<br>Mean: No	542 (case)

Study (BiGS)				descriptives	
GlaxoSmithKline (GSK)		NA	18-84 [47.1, 12.2]	Assuming 2003, 1919-1985<br>Mean: 1956 (2003)	899
Pritzker Neuropsychiatric Disorders Research Consortium	NIMH Repository	1991-1998	14-88 [42.2, 12.6]	1903-1984<br>NIMH Mean: 1953	1177 TOTAL CASE
	Michigan Repository	2005 (Not sure if DNA collected previous)		Michigan Mean: 1963	
Systematic Treatment Enhancement Program for Bipolar Disorder (STEP1)		1999-2003	>18	NA-1985<br>Mean: NA	922
Systematic Treatement Enhancement Program for Bipolar Disorder (STEP2)		NA/PROP UNPUBLISHED	NA	NA<br>Mean: NA	659
Thematically Organized Psychosis Study (TOP)		NA	NA	ND<br>Mean: NA	
Trinity College Dublin		NA	ND	ND<br>Mean: NA	
University College London (UCL)		1991-2006	NA	ND<br>Mean: NA	
University of Edinburgh		NA	ND	ND<br>Mean: NA	
Wellcome Trust Case Control Consortium (WTCCC)		1996	29-96	1900-1967<br>Mean: NA	1868 CASE
Cardiff UK		1990-2003	[44.8, 13.1]	ND<br>Mean: 1952 (Rolling, midpoint)	472

Clinical Antipsychotic Trials of Intervention Effectiveness (CATIE)	2001-2005	18-65 [41.3, 11.4]	1936-1987<br>Mean: 1962	402
ISC-Aberdeen	1997-2006	[40, 13.5]	ND<br>Mean: 1961	720
ISC-Cardiff	1999-2005	[36.8, 10.5]	ND<br>Mean: 1966	527
ISC-Dublin	2000-2005	[45.4, 11.7]	ND<br>Mean: 1958	270
ISC-Edinburgh	1986-2006	[42.2, 13.5]	ND<br>Mean: 1954	368
ISC-London	1983-2006	[45.3, 14.8]	ND<br>Mean: 1950	518
ISC-Portugal	1995-2002	[39.9, 14.7]	ND<br>Mean: 1959	346
ISC-SW1	2005-2009	[52, 11.5]	ND<br>Mean: 1955	168
ISC-SW2	2005-2009	[55.8, 12.8]	ND<br>Mean: 1952	390
MGS	1989-2008	[43, 11.6]	ND<br>Mean: 1954	2679
SGENE-Bonn	1992-2002	[34.1, 11]	ND<br>Mean: 1963	474
SGENE-Copenhagen	2003-2009	[41.3, 12.7]	ND<br>Mean: 1965	482
SGENE-Munich	1997-2010	[37.9, 11.7]	ND<br>Mean: 1967	434

SGENE-TOP3	2003-2007	18-65 [34.1, 10.2]	1938-1989<br>Mean: 1971	248
SGENE-UCLA	1995-2003	[34.8, 13.9]	ND<br>Mean: 1965	704
Zucker Hillside	1999-2006	[38.8, 10.4]	ND<br>Mean: 1964	192

Table S11: Birth cohorts for discovery samples--Coronary ARteryDIsease Genome wide Replication and Meta-Analysis (CARDIoGRAM) consortium, cardiovascular disease[10]

PERCENT TOTAL SAMPLE, BIRTHYEAR ASCERTAINED: 11.4%
WEIGHTED AVERAGE BIRTHYEAR: 1949.408

Study	Year(s)	Ages [mean,sd]	Birth Cohort	N
Atherosclerotic Disease Vascular Function and Genetic Epidemiology (ADVANCE)	Baseline 2001-2003	[45.8, 6.2]	ND<br>Mean: 1957	278
Atherogene Registry	1996-1997 Baseline<br>Registry	[Without Event-60.9, 10.1; With Event-67, 7.8]	ND<br>Mean: 1936 No MI; 1929 MI	2078
The Gutenberg Heart Study (GHS)	2007-2008	35-74 [55]	1933-1973<br>Mean: 1953	2078
Gutenberg Heart Express Study (GHSExpress)	2007-2008	35-74 [55]	1933-1973<br>Mean: 1953	2078
Age, Gene/Environment Susceptibility (AGES)	1967 Baseline		1907-1935<br>Mean: ND	
Atherosclerosis Risk in Communities (ARIC)	1987-1989	45-64	1923-1944<br>Mean: 1934	
Cardiovascular Health Study (CHS)	1989-1990	65-98<br>[72.3, 5.4]	1891 to approx. 1923-24<br>Mean: 1917	
Framingham Heart Study (FHS)	1948 baseline		Effectively 1886-1986<br>Mean: No wave data	
Rotterdam Study (RS)	1990-1993; 2000-2001 wave 2	>55 WI; >45 WII	ND-1956<br>Mean: Pooled/NA	
Diabetes Epidemiology: Collaborative Analysis of Diagnostic Criteria in	1997 Baseline	11.50-108 [m-64.74, f-57.94]	1889-1985<br>Mean: 1933	26799

Europe (deCODE)			(Males); 1940 (Females)	
German Myocardial Infarction Family Studies I (GERMIFS I)	1997-2002	32-82 [50.2, 7.8]	1915-1970<br>Mean: 1949	600
German Myocardial Infarction Family Studies II (GERMIFS II)	1997-2002	29-90 [51.4, 7.5]	1907-1973<br>Mean: 1948	1124
German Myocardial Infarction Family Studies III (GERMIFS III)	NO REF	[58.6, 8.7]	ND<br>Mean: NA	1157
Ludwigshafen Risk and Cardiovascular Health Study (LURIC 1)	1997-2000	[61, 11.8]	ND<br>Mean: 1937	
Ludwigshafen Risk and Cardiovascular Health Study (LURIC 2)	1997-2000	[63.7, 9.4]	ND<br>Mean: 1935	
MedStar	2004-2007	[48.9, 6.4]	ND<br>Mean: 1957	
Myocardial Infarction Genetics Consortium (MIGen)	Baseline 1997 (MGH; Europe indeterminate?)	<51 (male), <61 (female);<br><45 (Italian subsample, both sexes)<br>[42.4, 6.6]	1937-undefined<br>Mean: 1955 (MGH)	2,967 (+3075 control)<br>(2,647 TAG)
Ottawa Heart Genomics Study (OHGS)	NA	[48.7, 7.3]	ND<br>Mean: NA	
PennCATH	2009-2012	[52.7, 7.6]	ND<br>Mean: 1958	
Wellcome Trust Case Control Consortium (WTCCC)	1996	29-96	1900-1967<br>Mean: No	4862

descriptives

References (numbering scheme follows main article)

37. Stefanski, L.A., & Cook, J.R. Simulation-extrapolation: the measurement error jackknife. *J. Am. Stat. Assoc.* **90**, 1247-1256 (1995).
38. Yang, J., Lee, S.H., Goddard, M.E., &Visscher, P.M. GCTA: a tool for genome-wide complex trait analysis. *Am. J. Hum. Genet.* **88**, 76-82 (2011).
39. Ng, M., *et al.* Global, regional and national prevalence of overweight and obesity in children and adults 1980-2013: a systematic analysis. *Lancet* **384**, 766-781 (2014).
40. Lleras-Muney, A. The relationship between education and adult mortality in the United States. *Rev. Econ. Stud.* **72**, 189-221 (2005).
41. Domingue, B.W., *et al.* Genome-wide estimates of heritability for social demographic outcomes. *Biodemography Soc. Biol.* **62**, 1-18 (2016).
42. Colodro-Conde, L., Rijsdijk, F., Tornero-Gómez, M.J., Sánchez-Romera, J.F., &Ordoñana, J.R. Equality in educational policy and the heritability of educational attainment. *PLoS One* **10**, e0143796 (2015).
